# Melanosis of the urinary bladder with associated urinary tract infection

**DOI:** 10.1016/j.eucr.2025.102954

**Published:** 2025-01-16

**Authors:** Lauren Nesi, Jordan Sarver, Paramjot Gogia, Ali Baydoun, Barrett Anderson, Dongping Shi, Mazen Abdelhady

**Affiliations:** a Department of Urology, Detroit Medical Center, Detroit, MI, USA; b Department of Health Sciences, Brock University, St. Catharines, ON, Canada; c Department of Pathology and Laboratory Services, Detroit Medical Center, Detroit, MI, USA

**Keywords:** Melanosis vesicae, Benign bladder finding, Aerococcus, UTI, Histopathology

## Abstract

Melanosis of the bladder is an exceedingly rare benign condition that can mimic more serious urothelial pathologies. Here, we analyze the clinical presentation, associated symptomatology, and follow-up of a patient presenting with melanosis and associated urinary tract infection. This patient is a 72-year-old male undergoing workup for gross hematuria, lower urinary tract symptoms, and recurrent urinary infections. We subsequently discuss two additional incidental findings of melanosis and associated urinary tract infection at our institution, and introduce novel context for this condition that currently lacks a standardized management protocol. To our knowledge, this is the first case series on melanosis vesicae.

## 1 Introduction

Melanosis of the bladder, alternatively known as melanosis vesicae, is a rare benign condition caused by abnormal melanin deposition in cells of the urothelium, not extending deeper than the lamina propria.^1^^,^^2^ To date, there are approximately 25–37 cases described in the literature, and the exact etiology of the melanotic deposition remains unknown.^1^^,^^3^^,^^4^ While it is regarded as a benign condition, it can mimic more serious pathologies,^3^ necessitating careful histopathological and immunohistochemical evaluation. We discuss clinical significance, pathogenesis, and general urologic considerations.

## 2 Case presentation

### 2.1 Incidental finding

The patient is a 72-year-old African American male who presented with painless gross hematuria, recurrent UTIs with Enterococcus and Aerococcus species, trilobar prostatic hypertrophy, and lower urinary tract symptoms (LUTS). His past medical history was significant for hypertension, hyperlipidemia, angina, and erectile dysfunction. The patient also has a history of tobacco and alcohol use, and no significant family medical history of genitourinary malignancy.

**Fig. 1:**
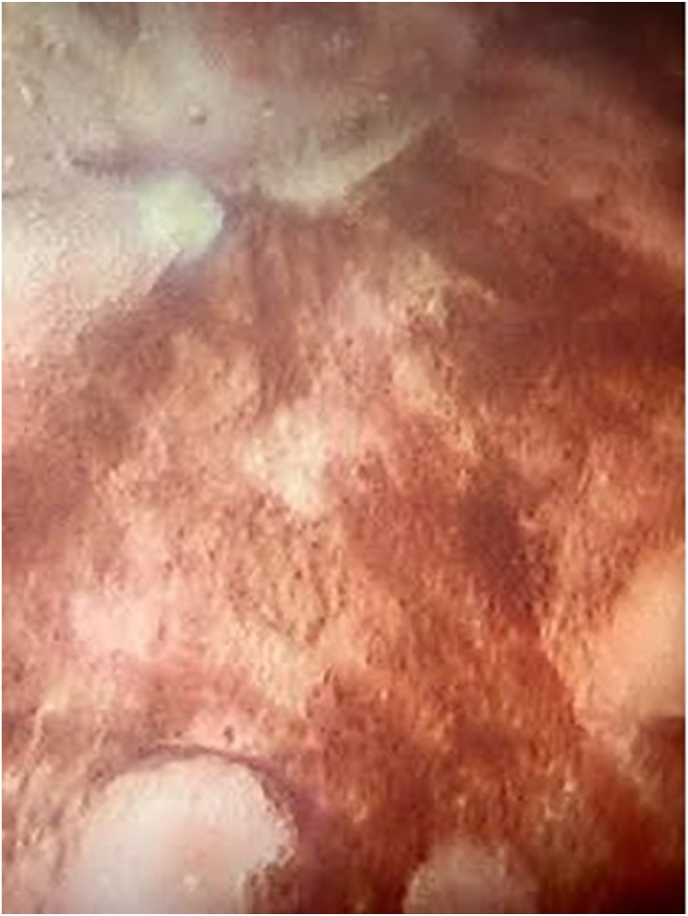
Gross imaging from cystoscopic examination of the posterior bladder wall significant for abnormal appearing black and velvety urothelial mucosa.

The physical examination revealed no costovertebral angle tenderness or suprapubic tenderness, and normal genitalia. Under cystoscopic examination in the office, the patient was found to have trilobar prostatic hypertrophy, two 1-cm bladder stones and an abnormal darkening of the bladder wall. Diffuse hyperpigmentation was seen on the bladder mucosa, but it was most notable on the posterior bladder wall, appearing black and velvety (Fig. 1).

### 2.2 Clinical course and follow-up

A repeat cystoscopy and endoscopic cystolitholapaxy were performed for stone extraction five months later, after the patient canceled multiple surgery dates. The melanotic lesions were stable and unchanged from the previous cystoscopic exam. No other melanocytic lesions were identified intraoperatively. Biopsies were obtained from darkly pigmented areas of the bladder wall. Approximately one more month later, the patient returned to our clinic, reporting a complete resolution of gross hematuria and voiding dysfunction.

### 2.3 Histopathological analysis

**Fig. 2:**
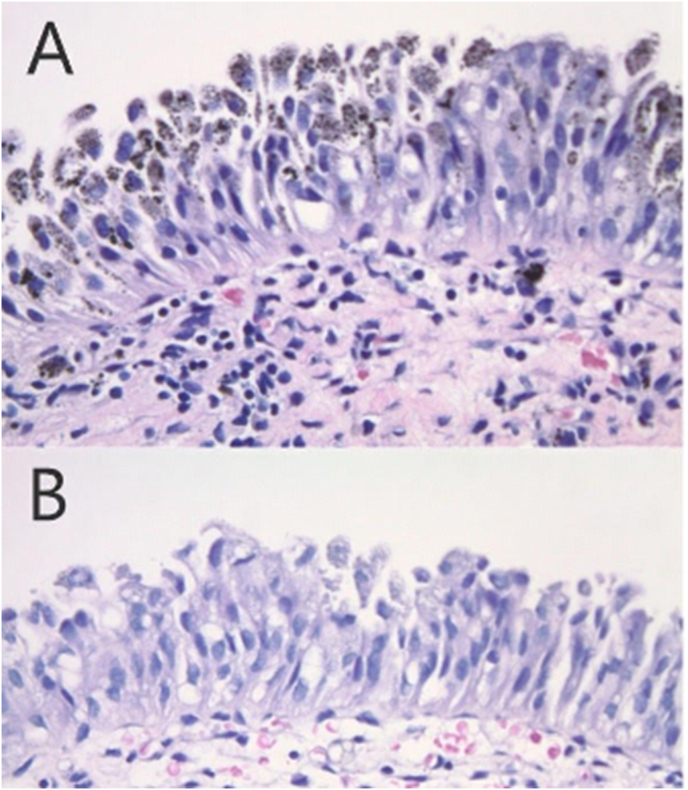
**A.** Microphotograph shows surface urothelium (i.e., urothelial mucosa) with intracytoplasmic dark brown to black pigments (Hematoxylin and eosin (H&E) stain; 400× magnification). **B.** Subsequent microphotograph shows the disappearance of intracytoplasmic pigment granules following bleach treatment for melanin (H&E stain; 400× magnification).

Two tan-pink soft tissue samples measuring 0.3 cm and 0.4 cm were sent for pathologic analysis (Fig. 2). The biopsy revealed urothelium with intracytoplasmic dark pigments negative for Periodic Acid Schiff, Human Melanosome 45, SOX10, p53, and Gomori's iron stain. The pigments were positive for the Fontana stain, consistent with melanin. The pigmentation also disappeared after treatment with bleach, and both samples were negative for carcinoma. These findings were pathognomonic for benign melanosis.

## 3 Discussion

Melanosis of the urinary bladder is an exceedingly rare benign condition that is scarcely covered in the literature. Melanosis is characterized by urothelial cell hyperpigmentation associated with abnormally increased melanin deposition in the cells, typically presenting as a velvety black urothelial discoloration.^5^ This condition is more frequently observed in anogenital skin, conjunctiva, and colorectal mucosa, and has a notably rare occurrence in the urinary bladder.^5^ The pathogenesis of urinary bladder melanosis remains unclear largely due to the unknown source of melanin granules, as melanocytes are not normally present in bladder urothelium. The prevailing hypothesis proposes that melanin granules may originate from melanocytic cells, which either differentiated abnormally from urothelial stem cells or migrated atypically from the neural crest in embryonic development.^2^ Two more recently proposed pathogenic mechanisms for melanotic deposits suggest that they might result from either an abnormal reaction of the mucosal nerve plexus or from the plasticity of the urothelial bladder wall.^6^ Diagnosis of urinary bladder melanosis involves cystoscopy and histopathological confirmation. However, natural progression remains undefined, and there is currently no established management or follow-up protocol.

Past case reports document mostly Caucasian patients ranging between the ages of 43 and 90; these showed no associated urothelial atypia but did show an association with a diverse range of urologic symptoms including but not limited to dysuria, urinary frequency, hematuria, incontinence, retention, nocturia, urinary obstruction, malodorous urine, and recurrent cystitis.^3^^,^^7^ While reported nearly equally in both males and females, it shows a slight male preponderance and is more common in those over age 40.^2^ Though it is generally considered a benign condition, six cases of concurrent urothelial carcinoma have been described in the literature, with individual reports associating it with primary malignant melanoma of the urinary bladder.^3^^,^^4^^,^^7^, ^8^, ^9^, ^10^ The rate of concurrent urothelial malignancy accounts for 16 % of all reported cases of urinary bladder melanosis (insofar as the number of total reported cases is 37).^11^

Melanosis of the bladder remains an incredibly rare disease, and therefore it should be noted that two other patients were also incidentally seen at our institution with melanosis and associated UTI. The first of these additional patients has been discussed previously in an independent case report.^12^ The patient is a 72-year-old African American male patient who presented with left-sided abdominal pain. His past medical history was significant for LUTS and urinary retention. CT imaging identified a complex, multiloculated diverticulum on the right anterior lateral dome of the bladder and another diverticulum on the left posterior bladder dome. Urine culture findings indicated an infection with Aerococcus urinae (>100,000 CFU/mL), likely caused by urinary stasis from the bladder diverticulum. Given multiple prior episodes of urinary retention requiring chronic indwelling catheterization, the patient opted for cystoscopy and subsequent bladder diverticulectomy. The initial cystoscopy showed diffuse, black splotchy lesions throughout the bladder. The inside of the left lateral wall diverticulum also revealed dark, velvety areas of pigmentation in the urothelial mucosa. Biopsies of these lesions were obtained to rule out malignancy. Pathological analysis showed benign urothelium with intracytoplasmic and subepithelial dark pigments positive for Fontana Masson stain, consistent with melanin, but negative for PASd, iron stain (which cleared with bleach treatment), and melanocytic markers (HMB45, SOX10, S100), ruling out melanoma. Definitive surgical management was deferred until the biopsy specimens obtained during cystoscopy resulted. The patient subsequently underwent an open left lateral posterior bladder diverticulectomy. The final surgical pathology confirmed absence of malignancy in the excised diverticulum. Postoperatively, the patient recovered well without the need for further catheterization^12^.

The second additional patient seen at our hospital is a 23-year-old African American female with no known past medical or surgical history. She presented to the emergency department with chills, flank pain, right lower quadrant pain, dysuria, urgency, and frequency for the past week, with rapidly worsening symptoms immediately prior to the visit. The urinalysis showed signs of infection, and a CT of the abdomen and pelvis demonstrated a distal right ureteral calculus with upstream hydroureteronephrosis. The patient elected to undergo cystoscopy, right retrograde pyelography (RP), and right ureteral stent insertion for the obstructing calculus. During the procedures, there were two irregularly shaped, well-demarcated, dark, velvety, and pigmented melanotic lesions seen in the bladder mucosa on the posterior and right lateral walls. These lesions were accompanied by surrounding erythema, suggesting local inflammation. The texture of the lesions appeared smooth with no evidence of ulceration. No biopsies were taken at this time due to the benign appearance of the lesions, so no histopathology is available. There were no tumors, stones, or diverticula in the bladder. The bilateral ureteral orifices were in an orthotopic position, and no filling defects or hydronephrosis were noted during retrograde pyelography. However, a calcification was seen adjacent to the distal ureter and a stone could not be excluded. The patient had a ureteral stent placed. The final urine culture showed *Escherichia coli*, and she was treated with the appropriate antibiotic therapy. The patient was discharged home with a ureteral stent in place and subsequently followed up outpatient. Two months later, the patient elected to undergo definitive stone management with cystoscopy with ureteroscopy and laser lithotripsy. The cystoscopic exam showed that the bladder lesions were still apparent and unchanged. However, the ureteroscopy was negative for urolithiasis, and the stent was removed. The patient denied any further bothersome urinary symptoms at subsequent appointments.

The index patient reported in this study had bladder melanoma ruled out via histopathological evaluation. For the first additional case, bladder melanoma was ruled out via endoscopic biopsy, and no cystoscopic follow-up was performed to monitor the melanotic lesions after diverticulectomy.^12^ Notably, this previously reported case report is the first to document the aforementioned association between bladder melanosis within a bladder diverticulum; this confluence of symptoms may provide a novel context for urinary bladder melanosis^12^. In contrast, the second additional patient did not undergo a bladder biopsy. Although urologists may elect to biopsy melanosis due to the reported association with malignancy,^3^^,^^4^^,^^7^, ^8^, ^9^, ^10^ there is no established guideline recommendation to do so. Also, the last patient had no change in these lesions over the course of two months, seen during follow-up ureteroscopy.

Interestingly, both our patient and the first additional case seen at our institution had UTI caused by *Aerococcus* spp. This unusual aerobic gram-positive coccus genus includes multiple species—mainly A. urinae and A. sanguinicola—and is increasingly recognized as a uropathogen, especially in elderly and immunocompromised patients.^13^ Within the literature, there is only one other documented case of urinary bladder melanosis in a patient with bladder diverticula and a concurrent Aerococcus UTI..^14^ The potential relationship between Aerococcus infection and melanosis is not yet established; whether the identified organisms contribute to bladder pigmentation or are simply incidental concurrent findings remains to be understood.

Bladder diverticula create pouches that can retain urine, leading to stasis. Further, they are prone to complications such as infections and stone formation.^15^ Consistent with more recent theories on the pathogenesis of melanosis,^6^ it is possible that melanotic pigmentation on the walls of bladder diverticula may result from chronic inflammation due to urinary stasis and concurrent infection. Relatedly, bladder melanosis has previously been reported alongside neurogenic overactive bladder and focal chronic inflammation of the superficial lamina propria.^6^^,^^16^ However, further studies are needed to establish causality pertaining to whether these sustained inflammatory or immunologic stimuli promote melanogenesis.

Two individual benign cases of melanosis have demonstrated a complete and spontaneous resolution of the melanotic lesions upon cystoscopic follow-up.^1^^,^^17^ Hence, melanosis vesicae should be considered in the differential diagnosis of urothelial hyperpigmentation of the bladder. Despite the generally benign course, for cases of urinary bladder pigmentation or melanotic deposition with the diagnostic features presented in this case series, urologists should at least rule out concurrent urothelial malignancy. However, while bladder wall biopsy is reasonable when melanosis is suspected, there is no established guideline recommendation to do so. It should be noted that for our patient as well as the other two who were seen at our institution, the melanotic lesions were found to be stable and unchanged in size, morphology, and distribution at follow-up cystoscopic examination.

There is a paucity of data to provide any recommendations regarding regular cystoscopic surveillance. While the typically benign nature of this condition contrasts with its somewhat jarring gross appearance on cystoscopic examination, histopathological confirmation to exclude associated urothelial malignancy is reasonable. Nevertheless, the absence of discernible changes in the pigmented lesions seen during follow-up cystoscopy challenges the notion of vigilant monitoring.

To our knowledge, while urinary bladder melanosis (i.e., melanosis vesicae) has been previously reported in individual cases globally, this is the first case series published in peer-reviewed literature. Our report is particularly unique in that we describe a primary patient presenting with melanosis and associated UTI, as well as two other independent occurrences within the same metropolitan area and hospital system. Given the rarity of this condition and its limited coverage in literature, it is difficult to understand its risk factors and pathogenesis. Hence, longer-term surveillance and prospective studies are warranted to develop a standardized management approach.

## 4 Conclusion

This study reports an incidental finding of melanosis vesicae with concurrent UTI. Melanosis is a rare condition that can mimic more serious pathologies. While it is generally a benign condition, multiple past case reports have found concurrent bladder malignancy. Further research is warranted to establish a causal relationship between melanotic bladder lesions and UTI, urolithiasis, bladder diverticula, and/or a combination of these associated urologic conditions.

## CRediT authorship contribution statement

**Lauren Nesi:** Conceptualization, Investigation, Methodology, Writing – review & editing. **Jordan Sarver:** Conceptualization, Investigation, Methodology, Writing – review & editing. **Paramjot Gogia:** Conceptualization, Investigation, Methodology, Writing – original draft, Writing – review & editing. **Ali Baydoun:** Conceptualization, Investigation, Methodology, Writing – original draft, Writing – review & editing. **Barrett Anderson:** Conceptualization, Investigation, Writing – original draft, Writing – review & editing. **Dongping Shi:** Conceptualization, Investigation, Methodology. **Mazen Abdelhady:** Conceptualization, Investigation, Methodology, Writing – original draft, Writing – review & editing.

## Consent

Informed consent was duly obtained for the publication of this case series report and any supplementary images.

## Data availability

Additional case data are not publicly available to protect patient anonymity.

## Disclosures

The authors have no conflict(s) of interests to disclose.

## Funding

This research did not receive any specific grant from funding agencies in the public, commercial, or not-for-profit sectors.
